# Iatrogenic Pseudoaneurysm: An Uncommon Cause of Deep Vein Thrombosis

**DOI:** 10.7759/cureus.2375

**Published:** 2018-03-27

**Authors:** Muhammad Khalid, Ghulam Murtaza, Majd Kanaa, Vijay Ramu

**Affiliations:** 1 Department of Internal Medicine, East Tennessee State University, Johnson City, USA; 2 Cardiology, Division of Cardiology, East Tennessee State University, Johnson City, USA

**Keywords:** psuedoaneursym, femoral, thrombosis, venous thrombosis, vein thrombosis, pseudo-aneurysm

## Abstract

Femoral artery pseudoaneurysm (FAP) is a common complication associated with left heart cardiac catheterization. FAP is a pulsatile encapsulated mass usually formed three to seven days after removal of the arterial sheath post cardiac catheterization. Usually, FAP is asymptomatic. Groin pain and swelling are the most common complaints in symptomatic patients.  It can be associated with multiple different complications including rupture, bleeding, and vascular compression leading to venous thrombosis, limb ischemia, and neuropathy. Deep vein thrombosis (DVT) resulting from FAP is an unusual complication with very few cases reported in the literature. We present a case of right-sided DVT secondary to the compression of femoral vein resulting in venous outflow obstruction due to iatrogenic FAP post cardiac catheterization that was successfully managed conservatively.

## Introduction

Iatrogenic pseudoaneurysms (IPA) form when an arterial puncture site fails to seal, allowing arterial blood to jet into the surrounding tissues and form a pulsatile hematoma. Femoral artery pseudoaneurysm (FAP) occurs in 0.1% to 0.2% of diagnostic angiograms and 0.8% to 2.2% following interventional procedures [1]. Local manifestations of pseudoaneurysm result from compression of surrounding tissues that can result in neuropathy, ischemia, and venous obstruction that can result in deep vein thrombosis (DVT). We present a case of right lower extremity DVT due to the compression of the femoral vein secondary to iatrogenic FAP.

## Case presentation

**Figure 1:**
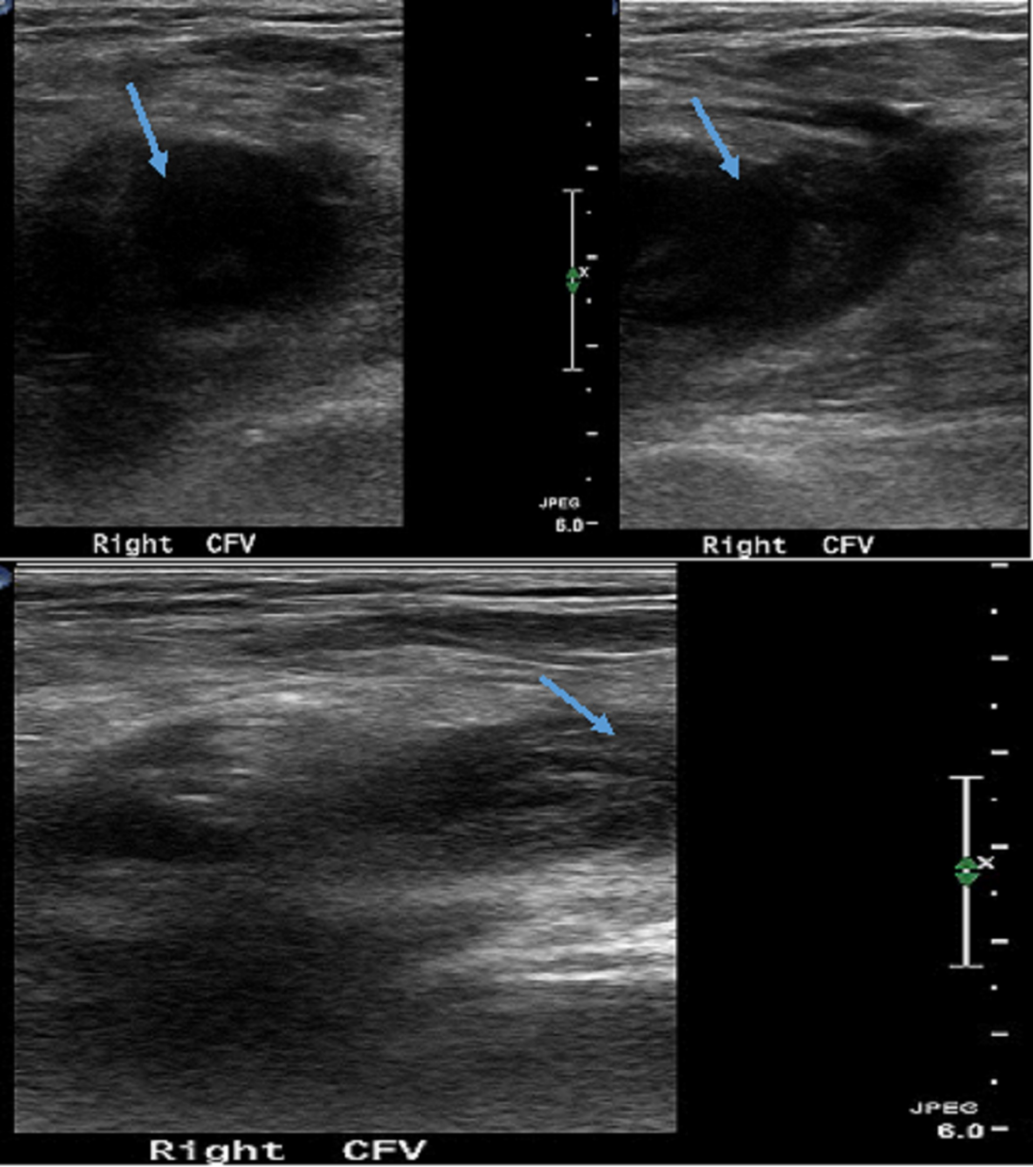
Doppler study showing thrombosis and non-compressible right common femoral vein (arrows)

A 78-year-old male with a medical history significant for hypertension, hyperlipidemia, and chronic smoking underwent evaluation for recurrent chest pain symptoms with coronary angiography. Access was obtained through the right femoral artery and six French sheaths were placed over the guide wire. Coronary angiography did not reveal any evidence of significant coronary artery disease. The sheath was removed without significant problems. A Mynx closure device was deployed and manual pressure was held for five minutes at the access site. The patient tolerated the procedure well and was discharged home the same day and recommended to follow up in the clinic. The patient returned to the clinic two weeks later with complaints of right groin pain, redness, and swelling that has been worsening for last one week. The patient reported that he had groin pain that started a week after the procedure which initially happened during exertion or ambulation and then progressed slowly at rest as well. Follow-up examination revealed a 2 cm mobile, pulsatile, and non-tender right groin mass in the instrumented area with associated bruising of groin and inner thigh and significant edema of the right leg. Lower extremity Doppler ultrasound was performed that showed DVT of the common femoral, superficial femoral and profunda femoral veins (Figure 1). 

**Figure 2:**
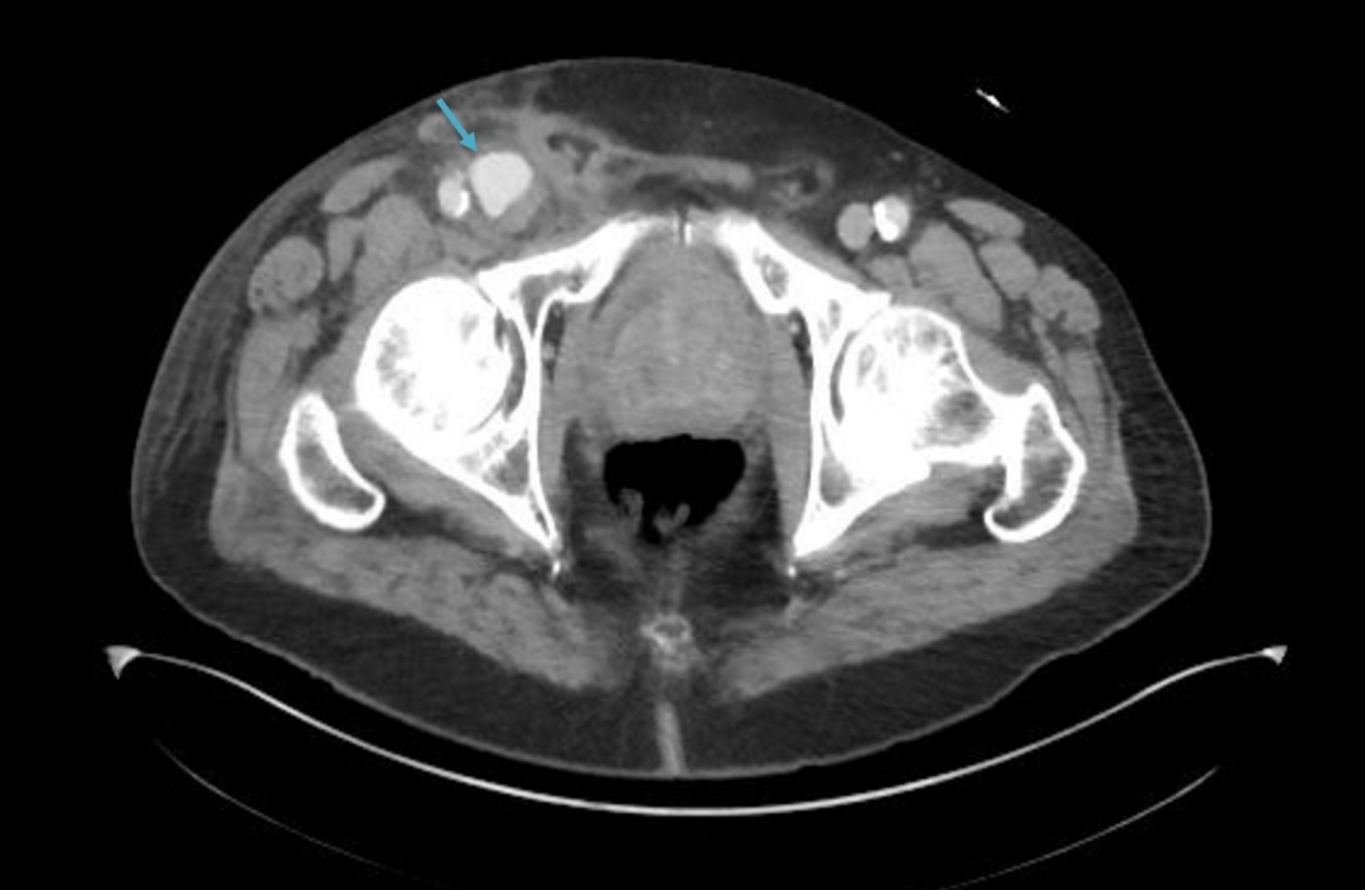
Computed tomography scan of the pelvis showing pseudoaneurysm of the right common femoral artery (arrow)

The patient did not report any history of trauma, intravenous drug abuse, prolonged immobilization, family history of blood clots, and recent travel. The patient had computed tomography (CT) scan of the chest, abdomen, and pelvis to further evaluate the etiology and extent of the thrombosis. A CT scan of the pelvis showed right groin pseudoaneurysm 3.1 cm x 2.8 cm. The pseudoaneurysm was compressing the femoral veins which were responsible for DVT in the right common femoral and superficial femoral veins (Figure 2).

The patient was started on low molecular weight heparin drip and evaluated by vascular surgery for thrombin injection the next day. Vascular surgery elected not to perform thrombin injection as pseudoaneurysm was found thrombosed in the vascular lab. The patient was started on apixaban for DVT for six months and instructed to wear a compression stocking and leg elevation to prevent stasis and complications of post-thrombotic syndrome. On follow-up evaluation one month later, the patient's symptoms had improved. The patient was recommended to continue apixaban and continue to follow up with the primary care provider.

## Discussion

FAP is a well-documented complication associated with femoral artery catheterization [2]. Pseudoaneurysms can be either iatrogenic or spontaneous [3]. It usually happens after three days of arterial sheath removal. Risk factors associated with pseudoaneurysm formation include old age, female gender, large size arterial sheath, inadequate manual compression, hypertension, peripheral vascular disease, hemodialysis, antiplatelet or anticoagulation use during an intervention, obesity, superficial artery cannulation, complex intervention, and bad puncture technique [4].

The possible etiology for DVT following cardiac catheterization is immobilization, prolonged manual compression at the groin site post catheterization, surrounding venous compression due to the hematoma or pseudoaneurysm [5-7]. Our case is a rare, interesting, and unique presentation of DVT most likely due to venous compression by a large 3 cm FAP, as we ruled out other factors. The patient had manual pressure for only five minutes and was discharged home on the same day. The patient denied immobilization, history of blood clots, and there was no hematoma or signs of infection or tumor-like osteochondromas on imaging. Only three case reports of DVT has been reported in the literature due to venous compression by pseudoaneurysm [8-10].

The clinical presentation can be asymptomatic as an incidental finding. Groin pain and leg swelling are the most common presentation in pseudoaneurysm with DVT. Our patient presented with groin pain and swelling. The groin examination is important to look for local complications associated with femoral artery catheterization. The three most important clinical signs are expanding pulsatile mass, bruit, and tenderness over the catheterization site. If two out of three signs are present, the clinical suspicion for pseudoaneurysm is very high.

Diagnosis can be done on physical examination based on the features mentioned above. Diagnosis usually involves duplex ultrasound, CT scan, and CT angiography (arteriography and venography). CT angiography is the gold standard, while duplex ultrasound can be used to differentiate between a hematoma or AV fistula, look for anatomic details, and measure velocity in the neck or sac. The typical features of venous thrombosis on ultrasound are lack of compressibility, obstruction of venous return, and a hypoechoic or isoechoic signal.

Different management approaches for pseudoaneurysm include conservative management, ultrasound-guided compression or thrombin injection, percutaneous thrombin injection, endovascular stent and surgical management, while the management of associated DVT is usually anticoagulation. In our case, pseudoaneurysm was impinging on nearby structures and was large enough to cause an external compression effect, significant enough to lead to DVT. Thrombin injection could have led to further inflammation and scarring of an already thrombosed pseudoaneurysm further worsening the DVT; hence, we decided not to proceed with the thrombin injection. The patient was continued on oral anticoagulation and he improved on follow up without further enlargement of pseudoaneurysm or associated DVT.

## Conclusions

FAP is a commonly reported complication associated with cardiac interventions. DVT due to venous compression secondary to FAP is very rare. Only few case reports have been reported in the literature. Our patient had a 3 cm pseudoaneurysm and DVT of the right leg due to venous compression by an aneurysm, diagnosed on Doppler US, managed conservatively, and discharged on oral anticoagulation for DVT. This case highlights the importance of early recognition of this uncommon presentation of DVT due to venous compression that could be managed conservatively.
